# ABC Transporter Subfamily E Is Critical for Gametogenesis and Eclosion in *Lygus hesperus* (Hemiptera: Miridae)

**DOI:** 10.3390/insects17050446

**Published:** 2026-04-23

**Authors:** J. Joe Hull, Evelien Van Ekert, Inana X. Schutze, Jeffrey A. Fabrick, Colin S. Brent

**Affiliations:** USDA-ARS U.S. Arid Land Agricultural Research Center, Maricopa, AZ 85138, USA; evelienvanekert@gmail.com (E.V.E.); inana.schutze@usda.gov (I.X.S.); jeff.fabrick@usda.gov (J.A.F.); colin.brent@usda.gov (C.S.B.)

**Keywords:** RNAi, ABC transporter, mirid, reproduction, oogenesis, spermatogenesis

## Abstract

Although the ATP-binding cassette (ABC) transporter superfamily of proteins typically facilitates the movement of compounds across cellular membranes, the ABC E subfamily (ABCE) influences protein synthesis via non-transporter roles in ribosome biogenesis. Despite this essential role, our understanding of the impact that ABCE proteins have on insect physiology is limited. Here, we identified and characterized the *ABCE* gene from *Lygus hesperus*, a major agricultural pest of crops in North America. *LhABCE* transcripts were constitutively expressed throughout development and were present in all adult tissues tested. RNA interference (RNAi)-mediated knockdown of *LhABCE* transcripts in fifth instar nymphs resulted in high nymphal mortality and an incomplete molt. *LhABCE* knockdown in adults disrupted gametogenesis and reduced longevity. In females, oogenesis was impaired and oocytes did not progress beyond the pre-vitellogenic phase. In males, *LhABCE* knockdown reduced both spermatozoa abundance and male fertility. *LhABCE* knockdown, however, had little to no impact on hemolymph protein levels or the levels of circulating vitellogenin. Taken together, the results indicate that LhABCE is critical for the normal progression of processes like molting and gametogenesis that require coordinated bursts of protein synthesis and suggest that ABCE may play an important role in the mechanisms underlying those bursts.

## Simple Summary

The ATP-binding cassette (ABC) transporter superfamily typically mediates substrate transport across membranes. Members of the ABC E subfamily (ABCE), however, do not engage in active transport but rather play a role in protein biosynthesis by regulating ribosome biogenesis. Despite this essential role, reports of how ABCE function impacts insect physiology are limited. In this study, we used RNA interference methods to examine the role of ABCE in the western tarnished plant bug (*Lygus hesperus*), a piercing–sucking agriculture pest. We show that ABCE is essential for adults as well as gamete development in both sexes. Knockdown in females completely inhibited egg production, whereas knockdown in males reduced both spermatozoa abundance and male fertility. Knockdown also impacted long-term adult survival. No effects were seen on hemolymph protein levels or the levels of circulating vitellogenin.

## 1. Introduction

The *Lygus* genus (e.g., *L. hesperus*, *L. lineolaris*, *L. ellisus*, *L. pratensis*, and *L. rugulipennis*) of polyphagous pests have a broad geographical range and attack many economically important crops [1,2,3]. In the United States, *L. hesperus* and *L. lineolaris* represent the most important pest risks with *L. hesperus* predominantly found in the western states and *L. lineolaris* in the southeast [4]. Although currently managed with chemical insecticides, changes in pest management strategies and agroecological conditions have resulted in increased incidences of *Lygus* infestation [5,6,7]. Resistance to multiple classes of insecticides has further exacerbated the challenges of *Lygus* management [8,9,10,11]. In addition, the newly adopted genetically modified cotton that produces the *Bacillus thuringensis* (Bt) toxin Mpp51Aa2 (formerly Cry51Aa2) may have limited long-term impact on *Lygus* spp. as it is considered a non-high-dose product with incomplete neonate mortality [12,13,14,15]. As such, new approaches to *Lygus* pest management are needed.

Proteins in the ATP-binding cassette (ABC) superfamily typically couple ATP binding and hydrolysis with the transport of diverse molecules across cellular membranes [16,17,18]. Eight subfamilies (ABC A-H) have been identified in arthropods based on sequence similarity and domain organization [19,20,21]. Unlike other members of the superfamily, the ABC E subfamily (ABCE) lacks transmembrane domains, does not function in cellular transport, and typically consists of a single-copy gene, although multiple *ABCE* genes have been identified in some insect genomes [22,23,24]. ABCE function is conserved across taxa with the proteins playing critical roles in ribosome biogenesis and recycling. Specifically, ABCE couples ribosome assembly with protein translation by facilitating maturation of the 60S ribosomal subunit and using ATP hydrolysis to drive dissociation of the 40S and 60S subunits from the 80S ribosome [22,23,25,26]. ABCE dysfunction results in reduced protein translation efficiencies and slow-growth phenotypes [22,23]. Among insects, null mutations in the *Drosophila melanogaster ABCE* gene (*pixie*) are embryonic lethal, and less severe mutations result in slowed growth [27]. RNA interference (RNAi)-mediated knockdown of ABCEs in *Tribolium castaneum*, *Diabrotica virgifera virgifera*, and *Peregrinus maidis* resulted in high mortality and incomplete molting [28,29,30]. Although these lethal phenotypes are associated with loss of ABCE function, the specific underlying roles of ABCE proteins remain largely unknown in insects.

Here, we expand on early bioinformatics-based analyses of *L. hesperus* ABC transporters [31] and show that RNAi knockdown of the *L. hesperus ABCE* homolog (*LhABCE*) negatively impacts adult eclosion, adult gametogenesis, and long-term adult longevity. The absence of obvious effects on either global hemolymph protein levels or circulating vitellogenin suggests that processes dependent on coordinated bursts of protein synthesis are most susceptible to knockdown. This suggests that ABCE may play an essential role in the differentiation of cells driving molting and gametogenesis.

## 2. Materials and Methods

### 2.1. Insects

Insects used were from a *L. hesperus* laboratory colony maintained at the USDA ARS Arid Land Agricultural Research Center in Maricopa, Arizona. Nymphs and adults were reared on green beans (*Phaseolus vulgaris* L.) and an ad libitum artificial diet packaged in Parafilm M (Pechiney Plastic Packaging, Chicago, IL, USA) [32,33]. Insects were maintained under a 14L:10D h photoperiod at 27 ± 1 °C and 10–40% relative humidity as described previously [34]. Eggs were collected from adult cages in oviposition gel packs filled with 1.25% carrageenan (Sigma-Aldrich, St. Louis, MO, USA). Daily monitoring of cages ensured timely collection of individuals within 24 h of egg hatch, nymphal molt, and adult eclosion.

### 2.2. Bioinformatics

To identify the *LhABCE* cDNA sequence, *L. hesperus* transcriptomes (PRJNA284294, PRJNA238835, and PRJNA210219) were searched using ABCE/pixie protein sequences from three model insects (*D. melanogaster*, AAF50342.1; *Bombyx mori*, NP_001036911.1; and *T. castaneum*, XP_968009). Searches were performed with an *e* value set to 1*e*^−5^ and all hits were re-evaluated using BLASTx v2.17.0+ (https://blast.ncbi.nlm.nih.gov/Blast.cgi; accessed on 18 March 2025) and the non-redundant (nr) database. Protein domain predictions were made with HMMER v3.1 [35] and ScanProsite v2025_01 [36]. Transmembrane domain predictions were made using default settings in TOPCONS v2.0 [37]. The cellular localization of LhABCE was predicted using PSORT II (https://psort.hgc.jp/form2.html; accessed on 18 March 2025) [38], and WoLF PSORT (https://wolfpsort.hgc.jp/; accessed on 18 March 2025) [39]. Multiple sequence alignment of LhABCE with other representative insect ABCE proteins (Table S1) was generated using MUSCLE 5.1 [40] in Geneious Prime v2024.0.7 (Biomatters Ltd., Auckland, New Zealand).

### 2.3. Cloning LhABCE

Total RNA was isolated from individual adult *L. hesperus* females at 1 and 6 d post-eclosion using TRI Reagent Solution (Thermo Fisher Scientific, Carlsbad, CA, USA) and purified with an RNeasy Plus Mini kit (Qiagen, Germantown, MD, USA). Total RNA was treated with DNase I (New England Biolabs, Ipswich, MA, USA), and cDNAs were generated from 500 ng RNA using custom-made random pentadecamers (Integrated DNA Technologies, San Diego, CA, USA) and a SuperScript III First-Strand Synthesis System (Thermo Fisher Scientific). The complete 1827 bp *LhABCE* open reading frame (ORF) was PCR-amplified using ExTaq premix (Takara Bio USA Inc., San Jose, CA, USA) and full-length primers (Table S2) designed to GBHO01014425 [31]. Thermocycler conditions consisted of a denaturation step (2 min at 95 °C), an amplification step (33 cycles of 20 s at 94 °C, 20 s at 56 °C, and 90 s at 72 °C), and a final extension step (5 min at 72 °C). PCR products were separated by electrophoresis on 1.5% agarose gels using SYBR Safe (Thermo Fisher Scientific). The expected 1.8 kb PCR product was subcloned into a pGEM-T Easy vector (Promega Corp., Madison, WI, USA) and Sanger sequenced (Arizona State University DNA Core laboratory, Tempe, AZ, USA). The consensus sequence was deposited in GenBank (KU356753.1).

### 2.4. Reverse Transcriptase-PCR (RT-PCR) Transcriptional Profiling of LhABCE

To examine the developmental expression profile of *LhABCE* transcripts, total RNAs were isolated from pooled eggs (*n* = 10), pooled nymphs (1st instar, *n* = 10; 2nd instar, *n* = 10; 3rd instar, *n* = 5; 4th instar, *n* = 5; and 5th instar, *n* = 3), and individual adults (males and females collected 0, 3, or 7 d post-eclosion). For tissue transcriptional profiles, total RNAs were obtained from pooled 7 d-old adult tissues. Each biological replicate consisted of heads, *n* = 15; thoraces, *n* = 5; abdomens, *n* = 5; midguts, *n* = 5; hindguts, *n* = 5; paired lateral and medal accessory glands, *n* = 5; paired testes *n* = 5; and paired ovaries, *n* = 5. cDNAs were generated as described above using 500 ng DNase I-treated total RNA. RT-PCR was performed using SapphireAmp Fast PCR Master Mix (Takara Bio USA Inc., San Jose, CA, USA) and primers (Table S2) designed to amplify ~500 bp fragments of *LhABCE* and *L. hesperus actin* (GDHC01004191). Transcript profiling was assessed across five biological replicates. Thermocycler conditions consisted of a denaturation step (2 min at 95 °C), an amplification step (35 cycles of 20 s at 95 °C, 20 s at 56 °C, and 30 s at 72 °C), and a final extension step (5 min at 72 °C). PCR products were electrophoresed as described above. Representative *LhABCE* products of the expected size were subcloned and their sequence confirmed via Sanger sequencing (Arizona State University DNA Core laboratory). Gel images were acquired with an Azure 200 gel imager (Azure Biosystems, Dublin, CA, USA) and cropped using Adobe Photoshop 25.9.0 (Adobe Systems Inc., San Jose, CA, USA).

### 2.5. Transient Expression of LhABCE in Cultured Insect Cells

Overlap extension PCR was used to generate a carboxyl terminal chimera of LhABCE fused in-frame with the fluorescent protein mVenus [41]. The chimera was generated from validated plasmids using gene-specific primers (Table S2) and KOD Hot Start DNA polymerase (Millipore Sigma, Burlington MA, USA), as described previously [42]. The chimeric PCR product was cloned into the pIB/V5-His-TOPO TA insect expression vector (Thermo Fisher Scientific) and Sanger sequenced.

Plasmids (2 μg) harboring either *LhABCE-Venus* or *Venus* alone were transfected overnight at 27 °C into *Trichoplusia ni* (*Tni*) cells (Allele Biotechnology, San Diego, CA, USA) on 35 mm #1.5 glass bottom dishes (Matsunami Glass USA Inc., Bellingham, WA, USA) using Cellfectin II (Thermo Fisher Scientific). Transfection media was replaced the next day with Ex-cell 420 serum-free media (Thermo Fisher Scientific). At 48 h post-transfection, the maintenance media was replaced with IPL-41 insect media (Thermo Fisher Scientific) and cells were stained with NucBlue Live reagent (Thermo Fisher Scientific). Cells were imaged on an Andor Spinning Disk Confocal BC43 microscope (Oxford Instruments, Abingdon, UK) with a Plan Fluor 40× 1.30 oil objective (Nikon Instruments Inc., Melville, NY, USA). Images were processed in Imaris v10.2.0 imaging software (Oxford Instruments) and cropped using Fiji v1.54p [43].

### 2.6. Double-Stranded RNA (dsRNA) Synthesis

A 493 bp fragment corresponding to nt 1025–1517 of *LhABCE* was amplified from sequence validated plasmid DNA using primers containing a 5′ T7 promoter sequence (Table S2) and SapphireAmp Fast PCR Master Mix (Takara Bio USA Inc.). As a negative control, dsRNA targeting the full-length *enhanced green fluorescent protein* (*EGFP*) sequence was generated from plasmid DNA [44] using similar T7 promoter-containing primers (Table S2). Thermocycler conditions consisted of a denaturation step (2 min at 95 °C), an amplification step (25 cycles of 20 s at 95 °C, 20 s at 61 °C, and 30 s at 72 °C), and a final extension step (5 min at 72 °C). The respective dsRNAs were synthesized and purified using a MEGAscript RNAi kit (Thermo Fisher Scientific), as described previously [45]. Purified dsRNAs were quantified via Abs_260_ using a Take3 module of a Cytation 5 multimode plate reader (Agilent, Santa Clara, CA, USA).

### 2.7. Nymphal RNAi

Male and female fifth instar nymphs were immobilized on ice then injected with 250 nL dsRNA (750 ng/μL) using a manual dsRNA delivery system [44]. Each injection set consisted of the *LhABCE* dsRNA experimental group and two negative control groups (*EGFP* dsRNA and non-injection). Injections were replicated five times with 10–15 nymphs per treatment for a total of 50–75 nymphs assayed. Nymphs that died within 10 min of injection were removed. Survivors were maintained under standard rearing conditions in Huhtamaki waxed cups (Huhtamaki, De Soto, KS, USA) covered with a mesh screen and provided green bean (*P. vulgaris* L.) pods once every two days. Nymphal post-injection mortality was tracked daily until adult eclosion. Normal and impaired eclosion phenotypes were imaged using a Leica DFC425 camera attached to a Leica M165C microscope equipped with an LED ring light (Leica Microsystems, Buffalo Grove, IL, USA).

Knockdown was evaluated by reverse transcription–quantitative PCR (RT-qPCR) using cDNAs prepared from a subset of nymphs sampled at 2 d post-injection (dpi). Amplification was performed in a 15 μL reaction mixture containing 7.5 μL of KAPA SYBR Fast qPCR Master Mix (Fisher Scientific, Hampton, NH, USA), 6.2 μL of ddH_2_O, 0.7 μL cDNA, and 0.3 μL primers (10 μM) on a CFX96 real-time quantitative PCR System (Bio-Rad, Hercules, CA, USA). Thermal cycling conditions included an initial denaturation at 95 °C for 2 min, followed by 40 cycles of denaturation at 95 °C for 5 s and then 64 °C for 10 s. A melt curve analysis consisting of a 65 °C to 95 °C heat ramp (0.5 °C s^−1^) was subsequently performed to confirm the specificity of amplification. Relative transcript abundance was assessed using the modified 2^−ΔΔCT^ method [46,47], which accounts for differences in primer efficiencies. Samples were normalized to non-injected controls and expression was determined relative to *L. hesperus b tubulin 1* (GDHC01011532.1). Primer efficiencies were determined to be >90% based on standard curves generated from seven 1:10 serial dilutions of linearized plasmid DNA. Knockdown was assessed using five biological replicates consisting of two pooled nymphs per replicate. Three technical replicates were run for each biological replicate. Primers used are listed in Table S2.

### 2.8. Adult RNAi

Adults (<24 h post-eclosion) were injected with 500 nL dsRNA (1 μg/μL) using a Nanoject III Programmable Nanoliter Injector (Drummond Scientific Company, Broomall, PA, USA) as described previously [45]. After a 10 min recovery period, post-injection survivors were transferred to rearing tubs and maintained under standard rearing conditions. Mortality was assessed daily and post-injection longevity of the *LhABCE* and *EGFP* dsRNA-injected insects was tracked until mortality was 100%. Each longevity assay was replicated three times with 35 adults per sex per replicated group. Survival rates were assessed using a log-rank survival analysis in SigmaPlot 15 (Grafiti LLC, Palo Alto, CA, USA). Knockdown was evaluated by RT-qPCR at 5 dpi across five biological replicates (one adult per replicate) per treatment, as described above.

The effects of *LhABCE* knockdown on ovarian development were evaluated at 7 dpi across four biological replicates consisting of 20–25 females per replicated treatment (non-injected, *EGFP* dsRNA-injected, and *LhABCE* dsRNA-injected). Ovaries were dissected under an SZ61 stereomicroscope (Olympus, Center Valley, PA, USA) and development was scored as described in [48]. Ovaries with semi-transparent white oocytes were characterized as pre-vitellogenic. Ovaries with at least one elongated yellow oocyte showing evidence of opperculum chorionation were characterized as chorionated. Subsets of ovaries from each treatment were imaged on an SMZ18 stereomicroscope equipped with a Nikon D5-Ri2 camera and NIS Elements v5.2.102 (Nikon Instruments Inc.).

To determine the effects of *LhABCE* dsRNA dosage on ovarian development, newly eclosed females (<24 h post-eclosion) were injected with dsRNA concentrations ranging from 0.003 to 4000 ng/μL. Ovarian development was qualitatively assessed as described above at 7 dpi and compared with non-injected females from the same cohort. The effect of each *LhABCE* dsRNA concentration was assessed in at least 10 females.

To determine the impact of *LhABCE* knockdown on spermatogenesis, newly eclosed males (<24 h post-eclosion) were injected with *LhABCE* or *EGFP* dsRNAs (30 ng/µL) and then maintained under standard rearing conditions. At 7 dpi, when males are typically reproductively mature [48], testes were dissected and a single testis from every male was homogenized in 20 μL distilled water. Spermatozoa in 10 μL aliquots were manually counted using a Bright-Line hemacytometer (Hausser Scientific, Horsham, PA, USA) on a DMLB compound microscope (Leica, Deerfield, IL, USA) equipped with a 10× objective (N PLAN, 0.25 PH1; Leica). Each treatment group was replicated three times with 10 males per treatment per replicate.

To examine the effects of *LhABCE* knockdown on adult mating, newly eclosed (<24 h) adults of each sex were injected with *LhABCE* or *EGFP* dsRNAs (3 ng/µL for females or 30 ng/µL for males) and maintained under standard rearing conditions. At 7 dpi, males and females from each treatment group were paired in reciprocal crosses with virgin females or males of equivalent age in Petri dishes (60 mm × 15 mm) provisioned with a section of green bean. After 12–18 h, female mating status was assessed based on the presence of a spermatophore visible beneath the ventral abdominal cuticle [49]. For male mating, individual females that had been paired with dsRNA-treated males were provided oviposition packets. Egg hatch was assessed daily from individual oviposition packets until no new nymphal emergence was observed. Each treatment group was replicated three times with 20–25 adults of each sex per treatment per replicate. Because oocyte maturation did not occur in females injected with *LhABCE* dsRNA, reciprocal experiments assessing egg hatch with knockdown females and wildtype males were not performed.

To assess the levels of circulating hemolymph proteins and vitellogenin in control and knockdown females at 7 dpi injection, a hindleg was removed with forceps and a 1 μL pipette tip placed at the wound site and gentle pressure was applied to the abdomen. Extracted hemolymph (0.2–0.5 μL per female) was diluted into 40 μL MES-SDS running buffer. Hemolymph was collected from 12 individual females per treatment group. The ovary developmental state was assessed in all samples and the *LhABCE* knockdown phenotype was confirmed. Protein concentrations were determined for each hemolymph sample using a Micro BCA protein assay kit (Thermo Fisher Scientific), and 3 μg protein from each replicate was subjected to SDS-PAGE analysis using NuPAGE 10% Bis-Tris gels (Thermo Fisher Scientific) and 1× MES-SDS (Thermo Fisher Scientific) running buffer. Gels were stained with SimplyBlue Safestain (Thermo Fisher Scientific), destained overnight in MilliQ water with gentle shaking, and imaged on an Azure 200 gel imager (Azure Biosystems Inc., Dublin CA, USA). Densitometry of the vitellogenin bands was performed using Fiji v1.54p [43], with band intensities normalized to the mean value of the non-injected controls. The identity of the protein band corresponding to vitellogenin was validated by mass spectrometry. Briefly, hemolymph samples from three females were individually electrophoresed and bands of the expected size (217.4 kDa) were gel excised and subjected to LC-MS/MS analysis on an Orbitrap Fusion Lumos Tribrid (Thermo Fisher Scientific) mass spectrometer at the Arizona State University Mass Spectrometry Core Research Facility (Tempe, AZ, USA). Fragmentation ions were compared with those predicted for *L. hesperus* vitellogenin (JAQ17632.1).

### 2.9. Statistics

Unless otherwise stated, statistical analyses were performed in GraphPad Prism 10.3.1 (GraphPad Software LLC., Boston, MA, USA) by evaluating differences between experimental and control treatment groups. Where applicable, outliers were removed based on the ROUT method. Differences were evaluated via one-way ANOVA with Tukey’s multiple comparison test (*p* < 0.05). Statistical differences that did not assume a normal distribution were assessed using ANOVA with a Kruskal–Wallis test and Dunn’s multiple comparison test (*p* < 0.05).

## 3. Results

### 3.1. Bioinformatic Analyses

Four *LhABCE* transcripts were identified from a *L. hesperus* transcriptome, including three with the same 608 aa ORF (Table S3). The fourth transcript encodes a 106-amino acid fragment with the highest similarity to a protozoan ABCE. The presence of a single identifiable *ABCE* sequence in *L. hesperus* is consistent with most reports that *ABCE* is a single-copy gene; however, the possibility that additional *LhABCE* transcripts are conditionally expressed cannot be excluded. BLASTx searches of the non-redundant NCBI database with the consensus *LhABCE* coding sequence revealed the highest similarity to hemipteran sequences identified as ABCE/pixie (Table S4) with sequence identity ranging from 79 to 94% (Figure S1). Motifs (Figure S2) and protein domains (Table S5) characteristic of ABC transporters are present in the LhABCE sequence; no transmembrane domains were found (Table S5).

### 3.2. Molecular Cloning and Expression Profile

**Figure 1:**
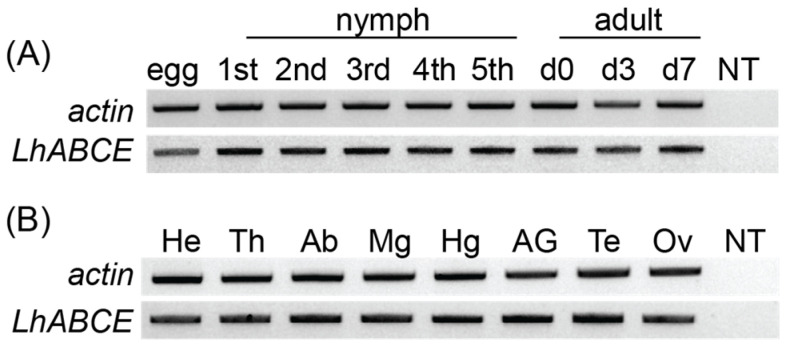
Constitutive expression of the *Lygus hesperus ABCE* transcript. (**A**) Developmental transcriptional profile. Partial transcripts for *actin* and *LhABCE* were amplified from eggs through all five nymphal instars (1st–5th) as well as mixed-sex adults at 0, 3, and 7 d post-eclosion (d0, d3, and d7). (**B**) Transcriptional profile in adult tissues. Transcripts were amplified from mixed-sex 7 d-old adult head (He), thorax (Th), abdomen (Ab), midgut (Mg), and hindgut (Hg) as well as accessory glands (AGs) and testes (Te) from males and ovary (Ov) from females. No template (NT). Gels shown are representative of results obtained across five replicates.

To confirm the transcriptomic sequence, the *LhABCE* ORF was amplified from whole adult female body cDNAs at different ages. Single amplicons of the expected size (1827 bp) were generated with all clones exhibiting > 99% nt identity with the transcriptomic sequence. We next used RT-PCR to assess *LhABCE* transcript abundance across development and in adult tissues. *LhABCE* was amplified in all developmental stages (Figure 1A) and all body segments and tissues examined (Figure 1B). This expression profile is consistent with a protein that fulfills a ubiquitous function such as the role reported for ABCE in protein synthesis.

### 3.3. Subcellular Localization of LhABCE

**Figure 2:**
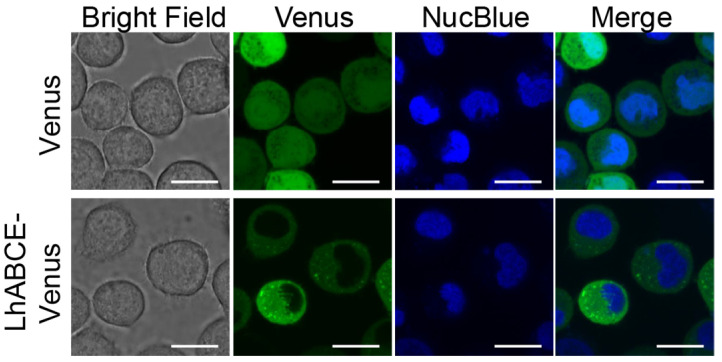
Subcellular localization of a *Lygus hesperus* ABCE fluorescent chimera in cultured insect cells. *Tni* cells were transfected with expression plasmids encoding Venus alone (green) or LhABCE-Venus (green). At 48 h post-transfection, cells were labeled with the nuclear marker NucBlue (blue) and then imaged. Scale bars = 20 mm.

Consistent with a role in protein synthesis, LhABCE was predicted to be cytoplasmic (Table S5). To test this prediction, we transiently expressed an LhABCE-Venus chimera (LhABCE fused in-frame with the fluorescent protein Venus) in cultured insect cells and examined its subcellular localization. Cells expressing Venus alone had diffuse fluorescence throughout the nucleus and cytosol (Figure 2). In contrast, fluorescent signals for the chimeric LhABCE protein were limited to the cytoplasm (Figure 2) and did not align with markers of endoplasmic reticulum, nucleus, nuclear envelope, peroxisome, lysosome, secretory pathway, Golgi network, or plasma membrane [50].

### 3.4. LhABCE Knockdown Effects on Adult Eclosion

**Figure 3:**
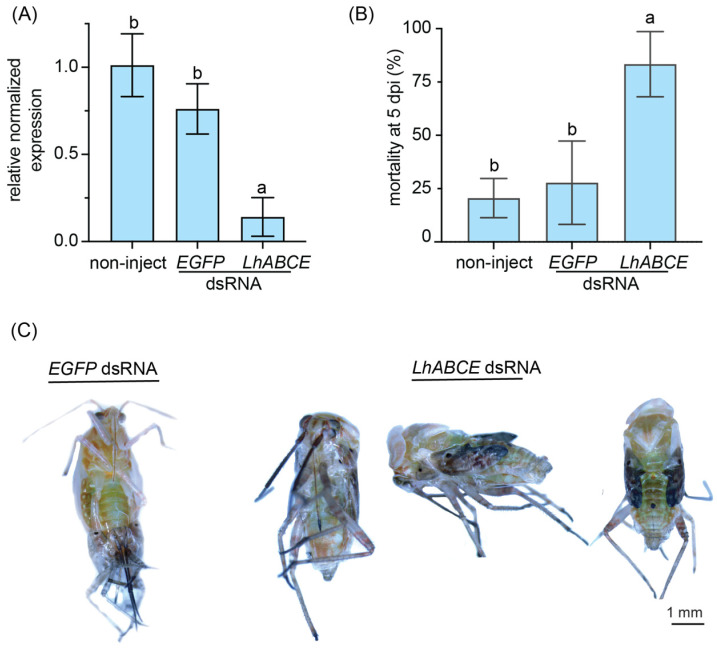
Effects of *LhABCE* knockdown in fifth instar nymphs. (**A**) RT-qPCR confirmation of *LhABCE* knockdown. Transcript abundance was assessed relative to *b-tubulin 1* in non-injected controls at 2 dpi. Bars represent the mean ± standard deviation (SD). Statistical differences are indicated by different letters (*p* < 0.0001; one-way ANOVA with Tukey’s multiple comparison test). Each treatment group, which consisted of two nymphs, was replicated five times. (**B**) Mean nymphal mortality at 5 dpi. Bars represent the mean ± SD. Statistical differences are indicated by different letters (*p* < 0.05; one-way ANOVA with Tukey’s multiple comparison test). The number of nymphs assessed per treatment: *n* = 70 for non-inject; *n* = 70 *EGFP* dsRNA; *n* = 70 for *ABCE* dsRNA. (**C**) Images showing incomplete eclosion in surviving *LhABCE* knockdown nymphs in contrast with normal adult eclosion in the *EGFP* dsRNA-injected group. All surviving nymphs from the controls underwent normal adult eclosion, whereas none of the nymphs from the *LhABCE* knockdown group completed eclosion.

To gain insights into the role LhABCE may have on the nymphal–adult molt, fifth instar nymphs were injected with dsRNAs targeting *LhABCE* or an RNAi control (*EGFP* dsRNA). At 2 dpi, *LhABCE* transcripts were significantly (*p* < 0.0001) reduced in the RNAi target group relative to either the *EGFP* dsRNA or non-injected group (Figure 3A). At 5 dpi, mortality in the *LhABCE* dsRNA group was significantly elevated relative to the control groups (*p* = 0.0102 vs. the *EGFP* dsRNA group; *p* = 0.0057 vs. the non-injected group). In contrast, no significant differences (*p* = 0.8358) in mortality were observed between the non-injected and *EGFP* dsRNA-injected groups (Figure 3B). Furthermore, those nymphs that survived in the *LhABCE* dsRNA group were unable to fully emerge from their exoskeleton and died during adult eclosion (Figure 3C). In contrast, all the nymphs in the control groups molted to adults.

### 3.5. LhABCE Knockdown Effects on Adult Gametogenesis

**Figure 4:**
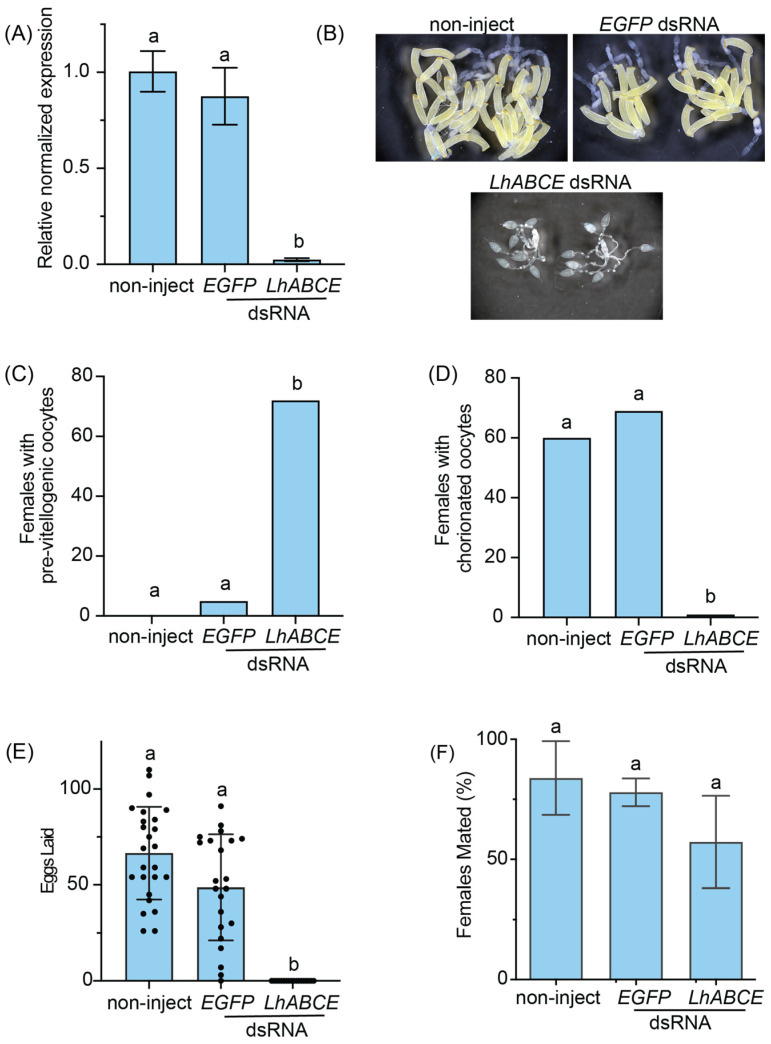
Effects of *LhABCE* knockdown on ovarian development and female fertility. (**A**) RT-qPCR confirmation of *LhABCE* knockdown. Transcript abundance was assessed relative to *b-tubulin 1* in non-injected controls at 5 dpi. Bars represent the mean ± standard deviation (SD). Statistical differences are indicated by different letters (*p* < 0.0001; one-way ANOVA with Tukey’s multiple comparison test). Each treatment group, which consisted of individual adults, was replicated five times. (**B**) Representative images of ovaries from non-injected, *EGFP* dsRNA-injected, and *LhABCE* dsRNA-injected *L. hesperus* females at 7 dpi. (**C**) Number of females with pre-vitellogenic ovaries at 7 dpi. Bars represent the mean ± SD. Statistical differences are indicated by different letters (*p* < 0.0001; Kruskal–Wallis with Dunn’s multiple comparison test). Knockdown was replicated four times with 20–25 females per treatment. The total number of females assessed per treatment across replicates: *n* = 60 for non-inject; *n* = 80 for *EGFP* dsRNA; *n* = 87 for *ABCE* dsRNA. (**D**) Number of females with chorionated ovaries at 7 dpi. Bars represent the mean ± SD. Statistical differences are indicated by different letters (*p* < 0.0001; Kruskal–Wallis with Dunn’s multiple comparison test). Knockdown was replicated four times with 20–25 females per treatment. The total number of females assessed per treatment across replicates: *n* = 60 for non-inject; *n* = 80 for *EGFP* dsRNA; *n* = 87 for *ABCE* dsRNA. (**E**) Individual female oviposition. Bars represent the mean ± SD of the number of eggs oviposited per female from 8 to 10 dpi. Statistical differences are indicated by different letters (*p* < 0.0001; Kruskal–Wallis with Dunn’s multiple comparison test). Knockdown was replicated three times with 10 females per treatment. The total number of females assessed per treatment across replicates: *n* = 25 for non-inject; *n* = 22 for *EGFP* dsRNA; *n* = 18 for *LhABCE* dsRNA. (**F**) Percentage of adult females that mated. Bars represent the mean ± SD. No statistical differences, indicated by different letters, were observed (*p* > 0.05; one-way ANOVA with Tukey’s multiple comparison test). Knockdown was replicated three times with 20–25 females per treatment. The total number of females assessed per treatment across replicates: *n* = 56 for non-inject; *n* = 39 for *EGFP* dsRNA; *n* = 37 for *ABCE* dsRNA.

Given the deleterious effects of *LhABCE* knockdown on molting, we next examined the effects of *LhABCE* knockdown on oogenesis and spermatogenesis. Adults < 1 d-old were injected with dsRNAs as before, and knockdown (*p* < 0.0001) of *LhABCE* transcripts was confirmed at 5 dpi (Figure 4A). By 7 d post-eclosion, *L. hesperus* ovaries are fully mature and typically contain multiple chorionated oocytes that have undergone vitellogenic expansion (Figure 4B). In contrast, ovarian development in *LhABCE* knockdown females was arrested with most oocytes pre-vitellogenic (Figure 4C; *p* < 0.0001) and few to no chorionated oocytes (Figure 4D; *p* < 0.0001). This developmental defect was specific to the *LhABCE* dsRNA group as the non-injected and *EGFP* dsRNA groups were comparable with no statistical differences (*p* = 0.2884) between the two (Figure 4C,D). Furthermore, from 8 to 10 dpi, the number of eggs oviposited by *LhABCE* knockdown females was significantly reduced (*p* < 0.0001) relative to the control groups, which were not statistically different (Figure 4E; *p* = 0.2834). Despite the absence of productive ovaries, *LhABCE* knockdown had no impact (*p* = 0.2690 vs. the *EGFP* dsRNA group; *p* = 0.1432 vs. the non-injected group) on the propensity of females to mate with wildtype males (Figure 4F). Surprisingly, ovarian development was remarkably sensitive to RNAi-mediated knockdown of *LhABCE*, as the arrested phenotype could be induced by sub-nanogram amounts of dsRNA (Table S6).

**Figure 5:**
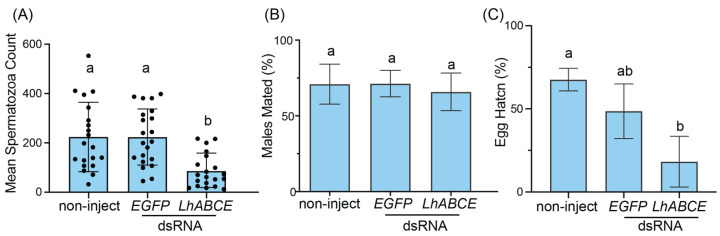
Effects of *LhABCE* knockdown on spermatogenesis and male fertility. (**A**) Spermatozoa abundance in individual *L. hesperus* adult males. Bars represent the mean ± standard deviation (SD) at 7 dpi. Statistical differences are indicated by different letters (*p* = 0.0011 for non-injected and *p* = 0.0004 for *EGFP* dsRNA; Kruskal–Wallis test with Dunn’s multiple comparison test). Knockdown was replicated three times with 10 males per treatment. The total number of surviving males assessed per treatment across replicates: *n* = 21 for non-inject; *n* = 21 for *EGFP* dsRNA; *n* = 21 for *ABCE* dsRNA. (**B**) Percentage of adult males that mated. Bars represent the mean ± SD. No statistical differences, indicated by different letters, were observed (*p* > 0.05; one-way ANOVA with Tukey’s multiple comparison test). Knockdown was replicated three times with 25 males per treatment. The total number of males assessed per treatment across replicates: *n* = 70 for non-inject; *n* = 65 for *EGFP* dsRNA; *n* = 73 for *ABCE* dsRNA. (**C**) Percentage of eggs from wildtype females mated with dsRNA-treated males that hatched. Bars represent the mean ± SD. Statistical differences are indicated by different letters (*p* = 0.01; one-way ANOVA with Tukey’s multiple comparison test). Knockdown was replicated three times with 25 males per treatment. The total number of males assessed per treatment across replicates: *n* = 55 for non-inject; *n* = 46 for *EGFP* dsRNA; *n* = 47 for *ABCE* dsRNA.

Spermatogenesis in males was likewise impaired following *LhABCE* knockdown. Spermatozoa abundance was significantly reduced relative to the *EGFP* dsRNA (*p* = 0.0004) and non-injected (*p* = 0.0011) control groups (Figure 5A). Despite this reduction, the propensity of *LhABCE* knockdown males to mate with wildtype females did not differ from either the non-injected (*p* = 0.8544) or *EGFP* dsRNA-injected (*p* = 0.8371) groups (Figure 5B). A significant (*p* = 0.01) reduction in hatch, however, was evident in eggs derived from females that had mated with *LhABCE* knockdown males relative to those from females mated with non-injected males (Figure 5C).

### 3.6. LhABCE Knockdown Effects on Adult Longevity

**Figure 6:**
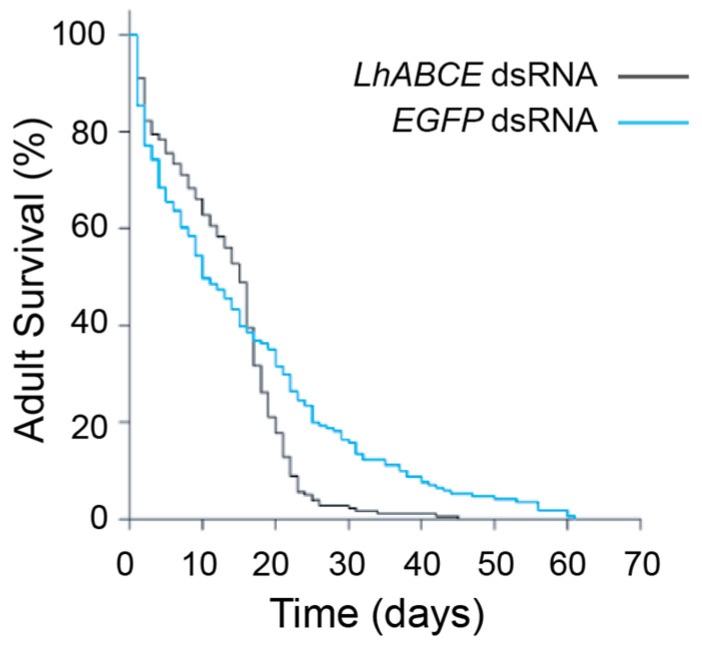
*LhABCE* knockdown effects on adult longevity. Survivorship probabilities for adult *L. hesperus* following injection of *LhABCE* or *EGFP* dsRNA. Major differences were observed between treatments in terms of late survivorship (*p* = 0.005; log-rank test, stat = 7.731; df = 1). The longevity assay was replicated three times with 35 adults per sex per replicated group.

*LhABCE* knockdown also impacted adult longevity (Figure 6; *p* = 0.005; log-rank test, stat = 7.731, d.f. = 1). Initially, survival curves for the *LhABCE* and *EGFP* dsRNA groups were comparable, with median survival times of 17 d and 19 d, respectively. In contrast, a pronounced increase in mortality was observed in the *LhABCE* knockdown group after 20 dpi, with third quartiles of 20 and 41 days, respectively.

### 3.7. LhABCE Knockdown Effects on Adult Hemolymph Proteins

**Figure 7:**
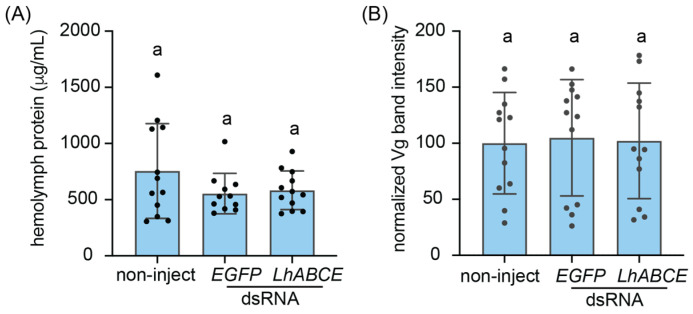
Effects of *LhABCE* knockdown on female hemolymph protein. (**A**) Circulating hemolymph protein levels. Bars represent the mean ± standard deviation (SD) at 7 dpi. No statistical differences, indicated by different letters, were observed (*p* > 0.05; Kruskal–Wallis test with Dunn’s multiple comparison test). The number of females assessed per treatment: *n* = 12 for non-inject; *n* = 12 for *EGFP* dsRNA; *n* = 12 for *ABCE* dsRNA. (**B**) Relative intensities of hemolymph vitellogenin (Vg) bands. Individual female hemolymph aliquots (3 µg) were subjected to SDS-PAGE on NuPAGE 10% Bis-Tris gels and subsequently stained with SimplyBlue Safestain. Densitometry analysis of bands corresponding to Vg was performed in Fiji and values were normalized relative to the mean of the non-injected Vg bands. Bars represent the mean ± SD at 7 dpi. No statistical differences, indicated by different letters, were observed (*p* > 0.05; one-way ANOVA with Tukey’s multiple comparison test). The number of females assessed per treatment: *n* = 12 for non-inject; *n* = 12 for *EGFP* dsRNA; *n* = 12 for *ABCE* dsRNA.

Given the pronounced effects of *LhABCE* knockdown on processes (i.e., molt and gametogenesis) characterized by bursts of protein synthesis and the established role of ABCE proteins in ribosome recycling, we sought to examine the impact of knockdown on general protein synthesis. Protein levels in hemolymph extracted from knockdown and control females at 7 dpi injection, however, did not differ (*p* > 0.5) across treatments (Figure 7A). We next examined the impact of knockdown on hemolymph levels of vitellogenin (Vg). Densitometry analysis of validated Vg bands (Table S7) from SDS-PAGE gels of hemolymph proteins at 7 dpi (Figure S3) likewise showed no statistical differences (*p* > 0.5) across treatments (Figure 7B). The absence of an effect on hemolymph Vg levels despite the arrested ovarian development phenotype indicates that Vg synthesis and secretion were not impacted by *LhABCE* knockdown. Taken together, these results suggest that the effects of *LhABCE* knockdown on oogenesis are not due to disruption of general protein synthesis but rather likely involve some other proteogenic process in cells comprising the ovary.

## 4. Discussion

While most ABC transporter superfamily members in insects have been extensively characterized [21,51], our understanding of the ABCE subfamily is limited to a few species. What is known, however, is largely consistent with the paradigm that ABCE is critical for protein synthesis as the phenotypes observed following ABCE disruption are associated with processes characterized by bursts of coordinated protein synthesis. Null mutations in the *D. melanogaster pixie* gene are embryonic lethal, whereas less severe mutations limit body size [27]. Transgenic *D. melanogaster* RNAi lines targeting *pixie* exhibit elevated larval mortality [52] or arrested ovarian development [53]. Similarly, RNAi knockdown of *ABCE* in *T. castaneum*, *D. virgifera virgifera*, and *P. maidis* result in elevated mortality and incomplete molting [28,29,30]. Despite no evidence for transporter activity, insecticide-induced expression of ABCE has been reported for some insects [54,55,56,57]. Although the mechanism for how increased ABCE activity might contribute to detoxification remains to be elucidated, the studies suggest that ABCE protein in some species may be pleotropic. In this study, we used RNAi to gain insights into how LhABCE impacts physiological processes dependent on bursts of coordinated protein synthesis. *LhABCE* knockdown caused severe disruptions in the nymphal–adult molt as well as adult gametogenesis and longevity. Taken together, the results are consistent with a role in protein synthesis and lay the groundwork for further exploration of LhABCE function.

Given the conserved role of ABCE in translation and the results of previous studies [28,29,30], we hypothesized that molting, which requires coordinated bursts of protein synthesis [58,59,60], would be impacted by disruptions to ABCE function. In support of this, none of the nymphs in the *LhABCE* knockdown group survived through the nymph–adult molt, with most unable to completely shed their nymphal exoskeletons. The consistency of lethal effects on juvenile insects suggest ABCE could have potential as a pest control target. However, before this can be considered in full, additional studies need to be done including: assessing ABCE function across a broader set of insects, evaluating the efficacy of different targeting methods (e.g., environmental RNAi), and considering the potential impact on non-target species.

In insects, components of the ribosomal protein machinery are markedly upregulated during oogenesis [61,62,63], and transcripts encoding ribosomal proteins are among the most abundant in pre-vitellogenic oocytes [64,65,66]. In *D. melanogaster,* ribosomal reduction causes infertility [53], and the knockdown of transcripts encoding proteins that govern ribosome biogenesis arrest oocyte development [67]. Similar ovarian defects were observed following knockdown of *regulator of ribosome synthesis* 1 (*RRS1*) and *ribosomal protein L32* in *Aedes aegypti* [63], and ribosomal protein knockdown in *N. lugens* [68]. Here, we show that *LhABCE* knockdown completely arrests oocyte development, suggesting it could play a role in regulating the expression of proteins necessary for oogenesis. While impaired ovarian development phenotypes have been reported following knockdown of transcripts encoding ABCB, ABCG, and ABCH subfamily members [28,29,69], those effects reflect typical ABC transporter activity rather than perturbations in the protein translation machinery.

Like oogenesis, spermatogenesis is a highly ordered process that requires significant protein investment in components of the protein machinery [70,71,72]. As such, depletion or knockdown of proteins in the ribosomal complex has been shown to cause sterility in male insects [73,74,75]. In *L. hesperus*, *LhABCE* knockdown negatively impacted spermatogenesis as both spermatozoa abundance and male fertility were significantly reduced. Unlike females, which had no mature oocytes, spermatozoa were only reduced in *LhABCE* knockdown males rather than abolished. This difference could indicate incomplete or transient RNAi-mediated knockdown with partial recovery of target transcripts in males effectively diluting the observed phenotype (e.g., reduction in spermatozoa abundance rather than complete cessation). Alternatively, the different effects of *LhABCE* knockdown on gametogenesis (complete vs. partial) might reflect temporal differences in gonad development. Ovaries are largely quiescent in newly emerged adult female *L. hesperus*, with the initiation of vitellogenesis typically occurring two days post-eclosion [48]. This developmental period aligns with the timing of dsRNA injections (i.e., newly emerged adults) and would allow for target transcript knockdown to occur prior to oocyte maturation. In contrast, spermatozoa are present in fifth instar *L. hesperus* nymphs [76] and testes of newly emerged males [48,77]. Because *L. hesperus* spermatogenesis occurs throughout adulthood, knockdown on the day of emergence would be expected to only impact post-emergence increases in spermatozoa. Consistent with this, a reduction rather than a total absence in spermatozoa abundance was observed in *LhABCE* dsRNA-injected males. The direct biological effect of the reduction in spermatozoa may be the reduced fertility observed in the knockdown males. Alternatively, *LhABCE* knockdown may have negatively impacted sperm quality, as qualitative sperm defects and infertility have been reported in *D. melanogaster* following RNAi-mediated depletion of the ribosomal proteins RpS3 and Pelo [74,78].

Although we observed high nymphal mortality and an inability of *L. hesperus* nymphs to molt to adults following *LhABCE* knockdown, short-term (over the first 20 dpi) post-emergence mortality in knockdown females was comparable to controls. The absence of early-adult lethality might reflect rapid recovery of ABCE transcripts to pre-knockdown levels or compensation from an *LhABCE* variant that is specifically expressed in adults. *LhABCE* transcript knockdown, however, was pronounced at 5 dpi and effects on ovarian development remained obvious at 7 dpi, indicating persistence of the knockdown effect. We found no evidence for gene duplication or expression of an adult-specific variant; however, final determination will require a chromosome-scale genome assembly. We posit that the differences in *LhABCE* knockdown lethality reflect the need for coordinated bursts of protein synthesis to drive cell differentiation, organ expansion, and exoskeleton renewal of the molt [79,80]. Aside from gametogenesis, these translation bursts are largely inconsequential in adults, and, therefore, the physiological impact of *LhABCE* knockdown may be minimal. In support of this, we found that knockdown had no effect on either total hemolymph protein levels or circulating Vg, indicating that general protein synthesis was not substantially impaired following *LhABCE* knockdown. While ABCE involvement is presumably needed to maintain cellular homeostasis in adults, we speculate that residual post-knockdown LhABCE is sufficient to facilitate the basal translation requirements of the cell. Consistent with this, minor mutations in *D. melanogaster pixie* result in slowed growth [27]. The marked reduction in the long-term longevity of adults injected with *LhABCE* dsRNA, however, might reflect the impact of knockdown on translation fidelity. Incomplete or impaired dissociation of ribosome machinery from mRNA can reduce translation efficiency and result in deleterious proteins. To mitigate this possibility, the cell utilizes various mRNA quality control mechanisms that involve ABCE [81]. Consequently, ABCE dysfunction could promote the accumulation and propagation of proteins with translation errors, a defect associated with reduced longevity [82,83]. Disruption of the ribosome biogenesis-associated protein DIMT1/CG11837 reduced the longevity of *D. melanogaster*, *T. castaneum*, *N. lugens*, *Anopheles stephensi*, and *Leptopilina boulardi* [84]. While impaired mRNA quality control represents an intriguing hypothesis for the observed longevity differences, the impact of *LhABCE* knockdown on translation fidelity remains to be determined.

In addition to a dependence on coordinated bursts of protein synthesis, the processes impacted by *LhABCE* knockdown rely on cell differentiation—germline stem cells (GSCs) in gametogenesis and imaginal disks/midgut epithelial stem cells in molting [79,80]. Stem cells have elevated ribosomal content and are particularly sensitive to perturbations in ribosomal biogenesis [53,67,85,86]. Tissue-specific knockdown of *pixie* in *D. melanogaster* ovarian cells impair germline differentiation and oocyte development [53]. Similar defects were generated following the disruption of transcripts encoding a subset of DExD/H box proteins (Aramis, Athos, and Porthos) critical for ribosome biogenesis [67], and loss-of-function mutations in the ribosome biogenesis-associated protein Musashi prevented germ cell formation in *D. melanogaster* testes [87]. While the molecular mechanisms and cellular basis of gametogenesis in *L. hesperus* remain to be elucidated, we hypothesize that the arrested ovarian development and reduced spermatozoa phenotypes generated following *LhABCE* knockdown may also stem from defects in differentiation.

## Data Availability

Data is available upon request. The consensus *LhABCE* sequence was deposited in GenBank (KU356753.1).

## Supplementary Materials

The following supporting information can be downloaded at: https://www.mdpi.com/article/10.3390/insects17050446/s1, Figure S1: Conceptual translation of the *Lygus hesperus* ABCE transcript. Figure S2: Amino acid sequence identity heat map of insect ABCE proteins. Figure S3: Representative SDS-PAGE gel image of hemolymph proteins at 7 dpi. Table S1: Accession numbers and species abbreviations for sequences in Figure S2. Table S2: Oligonucleotide primers. Table S3: Top tBLASTn hits of *Lygus hesperus* TSA datasets queried with ABCE proteins. Table S4: Top five BLASTx hits for the *Lygus hesperus* ABCE transcript. Table S5: Protein metrics and predicted protein domains for *Lygus hesperus* and *Drosophila melanogaster* ABCE proteins. Table S6: *LhABCE* dsRNA dose–response effects on ovarian development. Table S7: Top five proteomic hits for gel-excised adult female *Lygus hesperus* hemolymph protein bands.

## Abbreviations

The following abbreviations are used in this manuscript:

ABC	ATP-binding cassette
ABCE	ABC subfamily E
dpi	days post-injection
dsRNA	double-stranded RNA
EGFP	enhanced green fluorescent protein
ORF	open reading frame
RNAi	RNA interference
Vg	vitellogenin
