# Supplementary material for: ABC Transporter Subfamily E Is Critical for Gametogenesis and Eclosion in Lygus hesperus (Hemiptera: Miridae)
Source: Insects. 2026 Apr 23;17(5):446. doi: 10.3390/insects17050446 (PMC13207602; doi:10.3390/insects17050446)
Supplement: Supplementary file 1 [file insects-17-00446-s001.zip › Figure S1.pdf]

	DmABCE1	BmABCE1	TcABCE1	BtABCE1	**LhABCE1**	LsABCE1	HhABCE1	TuABCE1
DmABCE1		84.1%	86.2%	80.6%	84.0%	83.1%	83.7%	77.1%
BmABCE1	84.1%		85.9%	79.9%	83.8%	82.6%	84.4%	75.8%
TcABCE1	86.2%	85.9%		84.2%	88.2%	87.0%	89.5%	79.6%
BtABCE1	80.6%	79.9%	84.2%		86.9%	86.4%	86.9%	77.0%
**LhABCE1**	84.0%	83.8%	88.2%	86.9%		90.1%	93.9%	79.4%
LsABCE1	83.1%	82.6%	87.0%	86.4%	90.1%		89.8%	79.4%
HhABCE1	83.7%	84.4%	89.5%	86.9%	93.9%	89.8%		80.1%
TuABCE1	77.1%	75.8%	79.6%	77.0%	79.4%	79.4%	80.1%	
