# Supplementary material for: ABC Transporter Subfamily E Is Critical for Gametogenesis and Eclosion in Lygus hesperus (Hemiptera: Miridae)
Source: Insects. 2026 Apr 23;17(5):446. doi: 10.3390/insects17050446 (PMC13207602; doi:10.3390/insects17050446)
Supplement: Supplementary file 1 [file insects-17-00446-s001.zip › Figure S2.pdf]

1 10 20 30 40 50 60 70
MPRHKNQEESDKLTRIAIVNSDKCKPKRCRQECKKTCPVVRMGKLCIEVSPNDKIAAISEELCIGCGICVKKC

80 90 100 110 120 130 140
PFEAITIINLPSNLERDTTHRYSQNSFKLHRLPIPRPGEVLGLVGTNGIGKSTALKILAGKQKPNLGRYTSPP

150 160 170 180 190 200 210
DWTEILNHFRGSELQNYFTKILEDDLKALIKPQYVDQIPKAVKGTVQHLLDRKDERNNQNEICDLLDLDKIRD

220 230 240 250 260 270 280 290
RHIEDLSGGELQRFACAMVCIQDGDIFMFDEPSSYLDVKQRLNAAVTIRSLIRPDKFIIVVEHDLSVLDYLSD

300 310 320 330 340 350 360
FICCLYGVPGAYGVVTMPFSVREGINIFLDGFVPTENLRFRDESLVFKVAESATEEEVKRMQHYEYPNMSKTM

370 380 390 400 410 420 430
GNFKLTVDSGQFTDSEIVVLLGENGTGKTTFIRMLAGNLAPDDGSGDLPSLNISYKPQKISPKSTGLVRHLLH

440 450 460 470 480 490 500 510
EKIRDAYVHPQFVTDVMKPMKIDDIMDQEVQNLSGGELQRVAMALCLGKPADVYLIDEPSAYLDSEQRLVCAK

520 530 540 550 560 570 580
VIKRFILHAKKTGFVVEHDFIMATYLADRVIVFEGSPSVLTNANTPQSLLAGMNRFLELLQITFRRDPNNFRP

590 600 608
RINKANSVKDVDQKRAGQYFFLED
