# Supplementary figures and images for: ABC Transporter Subfamily E Is Critical for Gametogenesis and Eclosion in Lygus hesperus (Hemiptera: Miridae)
Source: Insects. 2026 Apr 23;17(5):446. doi: 10.3390/insects17050446 (PMC13207602; doi:10.3390/insects17050446)

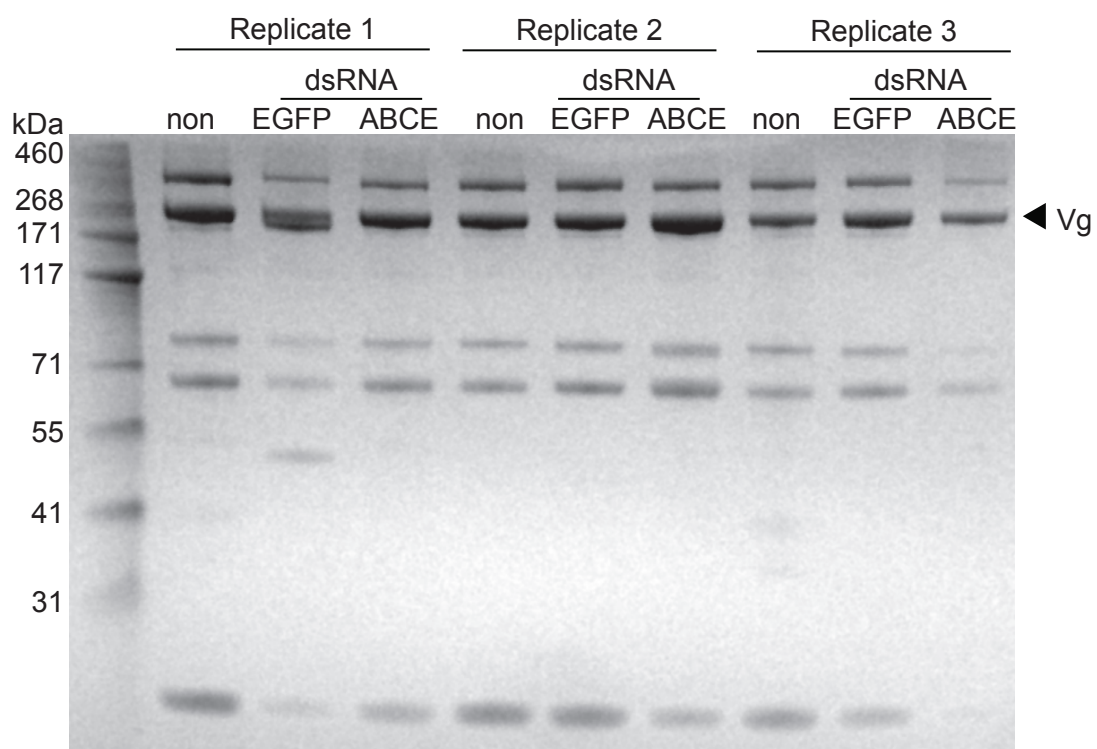

Supplement: Supplementary file 1 [file insects-17-00446-s001.zip › Figure S3.pdf]
